# SARS-CoV-2, Placental Histopathology, Gravity of Infection and Immunopathology: Is There an Association?

**DOI:** 10.3390/v14061330

**Published:** 2022-06-18

**Authors:** Leonardo Resta, Antonella Vimercati, Gerardo Cazzato, Margherita Fanelli, Sara Vincenza Scarcella, Giuseppe Ingravallo, Anna Colagrande, Sara Sablone, Mary Stolfa, Francesca Arezzo, Teresa Lettini, Roberta Rossi

**Affiliations:** 1 Section of Pathology, Department of Emergency and Organ Transplantation (DETO), University of Bari “Aldo Moro”, 70124 Bari, Italy; leonardo.resta@uniba.it (L.R.); margherita.fanelli@uniba.it (M.F.); sara.scarcella@policlinico.ba.it (S.V.S.); giuseppe.ingravallo@uniba.it (G.I.); anna.colagrande@gmail.com (A.C.); mary.stolfa@policlinico.ba.it (M.S.); teresa.lettini@uniba.it (T.L.); roberta.rossi@policlinico.ba.it (R.R.); 2 Section of Ginecology and Obstetrics, Department of Biomedical Sciences and Human Oncology (DIMO), University of Bari “Aldo Moro”, 70124 Bari, Italy; antonella.vimercati@uniba.it (A.V.); francesca.arezzo@uniba.it (F.A.); 3 Section of Legal Medicine, Department of Interdisciplinary Medicine, Bari Policlinico Hospital, University of Bari “Aldo Moro”, 70124 Bari, Italy; sara.sablone@policlinico.ba.it

**Keywords:** placenta, SARS-CoV-2, ACE-2, immunohistochemistry, CD4, CD8, RT-PCR, immunopathology

## Abstract

(1) Background: As the pandemic months progress, more and more evidence shows that the placenta acts as a “barrier” to SARS-CoV-2, although rare cases of vertical transmission have been described. (2) Methods: In an attempt to investigate whether the symptoms’ severity was related to different placental histological characteristics and the immune microenvironment, we subdivided 29 placentas from 29 mothers positive for SARS-CoV-2 into two groups, depending on the symptomatology (moderate/severe vs. asymptomatic/mild), performing immunohistochemical investigations for CD4 + and CD8 + T lymphocytes, as well as for CD68 + macrophage. We also evaluated the immuno-expression of the ACE2 receptor at the placental level. These two groups were compared to a control group of 28 placentas from 28 SARS-CoV-2-negative healthy mothers. (3) Results: The symptoms (likely to be related to viremia) were statistically significantly correlated (*p* < 0.05) with histopathological changes, such as maternal malperfusion, decidual arteriopathy, blood vessel thrombus of fetal relevance. Furthermore, the immuno-expression of ACE2 was significantly lower in SARS-CoV-2-positive groups vs. control group (*p* = 0.001). (4) Conclusions: There is still much to study and discover regarding the relationship between SARS-CoV-2 and histological changes in placentas and how the latter might contribute to various neonatal clinical outcomes, such as prematurity.

## 1. Introduction

More than a year and a half after its inception, the severe acute respiratory syndrome-coronavirus 2 (SARS-CoV-2) pandemic continues to affect the entire globe, posing new challenges and new questions, as important pathogenic mechanisms go on being discovered [1,2]. It has now been demonstrated that SARS-CoV-2 can affect different organs, featuring different manifestations and clinical pictures [3,4,5,6]. Surely, the fundamental relationship between pregnancy and SARS-CoV-2 was partially studied at the start of the pandemic, but it began to be more thoroughly investigated as new evidence came to the fore [7]. Even if much remains to be understood regarding the materno-fetal (vertical) transmission of SARS-CoV-2, if it does actually occur, a “barrier” role exerted by the placenta against potential placental infection, fetal transmission and the development of more or less overt disease due to SARS-CoV-2 (COVID-19) has been more clearly defined [8].

In the previous work, we illustrated some more frequent features in the placentas of infected women, in particular, maternal and deciduous thrombosis, increased intervillous fibrin and, in rare cases, fetal thrombosis [7].

In this work, we take a step forward, attempting to correlate the symptomatology of SARS-CoV-2-positive mothers with the extent of histopathological changes in the placental parenchyma. Here, we investigate the possible relationship between the symptoms’ gravity of SARS-CoV-2-positive mothers and the pattern of histological alterations, and we probe the ACE2 receptor immune expression.

## 2. Materials and Methods

### 2.1. Patients

The study was conducted on 29 placentas from 29 SARS-CoV-2-positive pregnant mothers followed at the Gynecology and Obstetrics Operative Unit from 31 October 2020 to 31 August 2021, identified through electronic clinical records. They were classified into two groups, based on the need for admission to the intensive care unit (COVID intensive care, CIC, vs. COVID not intensive care, CNIC). All women who presented during labor and delivery underwent testing, along with all neonates, with GeneXpert Dx Xpress SARS-CoV-2 RT-PCR (Cepheid, Atlanta, GA, USA) [9]. The analytical sensitivity and specificity of this test are reported by the manufacturers as 100% (87/87 samples) and 100% (30/30 samples), respectively, with a detection limit of 250 copies/mL or 0.0100 plaque-forming units per milliliter [10]. Positivity in the SARS-CoV-2 test was an independent criterion for the histopathologic analysis of the placentas. The clinical information, history and any medical therapies practiced were retrieved from the electronic medical records of Gynecology and Obstetrics. Symptoms considered mild included fever, cough, altered taste, malaise, headache, myalgia or muscle aches but not dyspnoea (breathing difficulties) or radiologically detectable changes.

Symptoms considered moderate included oxygen saturation (SpO2) equal to or greater than 94% and/or clinical or radiological evidence of pneumonia. Symptoms considered severe included SpO2 < 94% or respiratory failure (IR) signs/symptoms. For sampling of placental lesions, we adhered to the Amsterdam criteria [11], the parameters considered being: maternal malperfusion, decidual arteriopathy, fetal malperfusion, decidual inflammation, perivillous fibrin deposition, terminal villous hyperplasia, villous hypervascularization, thrombi in fetal vessels, syncytial nodes. In addition, we evaluated further placental alterations (not included in the Amsterdam criteria), namely choriamnionitis and perivillary histiocytosis. In order to study the distribution of immune cells in the groups under study, immunohistochemical investigations for CD4, CD8, CD68 and ACE2 were performed.

### 2.2. Controls

The CIC and CNIC groups were compared with a control group of 28 placentas from 28 healthy pregnant women with a physiological outcome, selected from historical controls [7], matched by gestational age and maternal age. All placentas in the control group were retrieved from birth cohorts prior to the onset of the coronavirus pandemic. Data were obtained from the electronic archives and from re-reading of the slides.

### 2.3. Procedure

The placentas were fixed in Formalin buffered at 10%, and photographs of the maternal and fetal surfaces were taken; they were then weighed, sampled and examined along the cut surface. The samples obtained included 2 rolls of amnio-chorial membrane, at least 2 samples from the umbilical cord, 3 from the maternal surface, 2 full-thickness sections and representative samples of any lesions present. All samples were subjected to routine treatment, inclusion, 5 µm sectioning and hematoxylin–eosin staining (H&E). They were observed with an Olympus BX-51 Optical Microscope (New York, NY, USA) equipped with the Olympus DP80 image acquisition system. On each section of the placenta, immunostaining with antibody rabbit anti-SARS-CoV-2 spike S1 glycoprotein monoclonal antibody, Thermofisher (Waltham, MA, USA), rabbit, was performed, as well as antigenic unmasking heat-induced citrate buffer epitope retrieval, for enzymatic immunohistochemical (IHC) analysis, at pH 6, diluted 1:800. In addition, immunostaining with anti-CD4 was performed: mouse monoclonal Ab (mAb), code M7310, (DAKO, Carpinteria, CA, USA), dilution 1:50; anti-CD8: mouse monoclonal Ab (mAb), code NCLCD8-295, (Novacastra Laboratories Ltd., Newcastle, UK), dilution 1:50; anti-CD68 (PG-M1): mouse monoclonal Ab (mAb), code GA613, (Dako Agilent, Santa Clara, CA, USA), dilution 1:100; anti-CD34: mouse monoclonal Ab (mAb), code QBEnd 10, (Dako Agilent), dilution 1:250. Finally, we used the anti-ACE2-Receptor: mouse monoclonal Ab (mAb), code ab89111, (Abcam, Cambridge, UK), dilution 1:250 [12].

The immunohistochemical reactions were evaluated, investigating the cell density for the CD4 and CD8 markers by counting positive cells in 10 fields (HPF) for each clinical case, at the level of the intervillous space and the maternal decidua (combined). Each field was examined at 400× magnification; the size of each field was 140 microns in length by 110 microns in width, the total amplitude of the field being 15,400 microns squared.

Assessment of ACE2-Receptor expression was performed by highlighting the chromogen signal on the cell membrane of maternal decidua [13] using the following score: grade 0 = no staining; grade 1 = weak staining; grade 2 = moderate staining; grade 3 = intense staining plus the score for the percentage of extension of the mass (score 0: <1%; score 1: 1–25%; score 2: 26–50%; score 3: 51–74%; score 4: >75%). The final score (sum of the 2 previous scores) was considered “High” if >3 vs. “Low” if <3.

All cases were examined independently under double-blind conditions by two pathologists with expertise in the field of perinatal pathology to confirm the diagnoses.

### 2.4. Statistical Analysis

A retrospective observational study was conducted on COVID-affected pregnant women to evaluate whether the gravity of the disease, evaluated by the need for admission to the intensive care unit, could be a risk factor for placental findings. A group of placentae randomly selected from a population of pregnancy with a physiological outcome was employed as the control group. A preliminary description of maternal features in the CIC, CNIC and control groups was produced. Comparisons of gestational age and placental weight among groups were performed with one-way analysis of variance (ANOVA) for independent groups, while the expressions of CD4, CD8, CD68 and ACE2 were compared among groups using the Kruskal–Wallis test for independent groups, as the Kolmogorov–Smirnov test showed a non-normal distribution. Post hoc test was employed for multiple comparisons. Maternal/pregnancy features and placental findings in the three groups were compared with the chi-square test. The odds ratio with 95% confidence intervals were calculated to evaluate the risk of specific placental injury in the CIC group compared to the CNIC group; when OR was not applicable, RR was calculated. To correct for the potential presence of type I errors induced by multiple testing, all results were corrected for false discovery rates (FDR) with alpha = 0.05. Quantities are reported as mean ± standard deviation. Categorical data are reported as frequencies and percentages. Statistical analysis was performed by means of SAS Software 9.4. (SAS Institute Inc., Cary, NC, USA).

## 3. Results

The maternal and pregnancy features are reported in Table 1. The mean gestational period was 35.2 ± 4.4 weeks for the CIC group, 36.9 ± 1.8 for the CNIC group and 38 ± 2 for the control group, with significant differences in gestational age among the groups (*p* = 0.02). Preterm births were more prevalent in the COVID groups compared to controls (*p* = 0.005). Among the 29 placentas analyzed, there were no cases of maternal–fetal transmission of SARS-CoV-2, neither in the intensive care group nor in the non-intensive care group. The other pregnancy features were equally distributed in the two groups (*p* > 0.05).

Regarding therapies in the CIC group of patients, one patient underwent heparin administration, while the remaining received symptomatic therapy with non-steroidal anti-inflammatory drugs (NSAIDs). Invasive ventilatory therapy was administered in seven patients, while six patients underwent non-invasive ventilation. In the CNIC group, seven women were asymptomatic and received no medications, while the remaining women, with mild symptoms, received symptomatic therapy based on NSAIDs.

The placental findings in the three groups are reported in Table 2. The mean placental weight was significantly different among the three groups, with lower values observed in the CIC group (*p* = 0.02). A higher percentage of maternal vascular malperfusion was observed in the CIC group compared to the CNIC group and control group (100% vs. 68.7% vs. 38.3%, *p* = 0.001), and the risk of maternal malperfusion was higher in the CIC group compared to the CNIC group (RR = 1.5, 95%CI: 1.1–2.2). The same applied for perivillous fibrin deposition (100% vs. 68.7% vs. 11%, *p* < 0.001), while the risk of this placental injury was not significantly higher in the CIC group compared to the CNIC group (RR = 1.4, 95%CI: 1–2). There were higher percentages of decidual arteriopathy (92.3% vs. 43.7% vs. 3.6%, *p* < 0.001) and fetal vessels thrombi (92.3% vs. 31.2% vs. 0, *p* < 0.01) in the intensive care group compared to the other groups. The risk of decidual arteriopathy, thromboses and terminal villous hyperplasia was higher in the CIC group than the CNIC group (O.R. = 15.4; 95%CI: 1.6–149; O.R. = 26.4; 95%CI: 2.6–262; O.R. = 6.9; 95%CI: 1.3–37, respectively). Instead, no higher risks in CIC than CNIC were observed in terms of fetal vascular malperfusion (O.R. = 1.4; 95%CI: 0.3–6.3), decidual inflammation (O.R. = 3.3; 95%CI: 0.5–20), villous hypervascularization (O.R. = 3.1; 95%C_I: 0.5–20.6), syncytial nodes (O.R. = 0.4; 95%CI: 0.06–2.5), chorioamnionitis (O.R. = 0.25; 95%CI: 0.02–2.6) and perivillary histiocytosis (O.R. = 4.28; 95%CI: 0.7–25). For these placental findings, except for villous hypervascularization and syncytial nodes, significant differences in both positive groups were observed vs. the control group.

**Figure A1:**
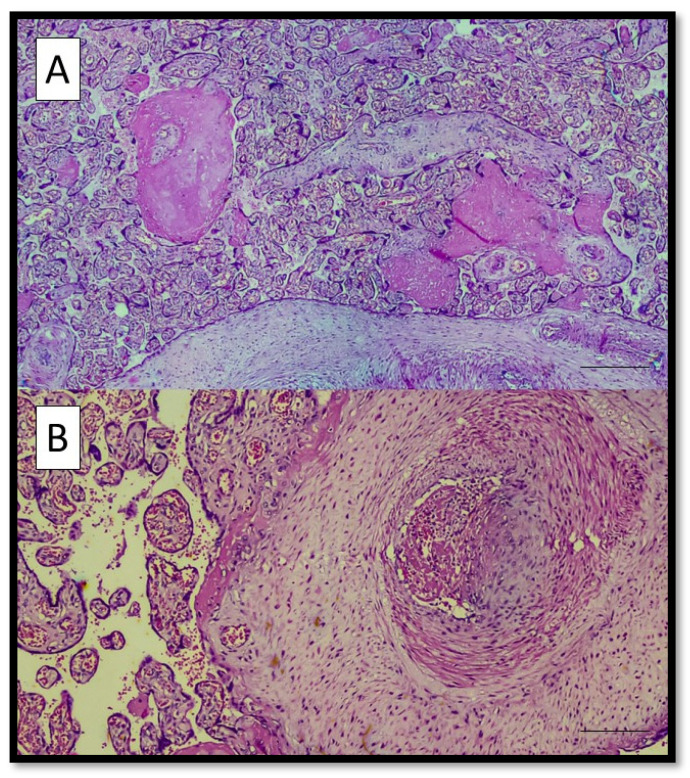
Placental findings in COVID intensive care, COVID not intensive care and control groups (**A**) Histological micrograph shows features of a chorionic disc of a SARS-CoV-2 positive placenta. Note perivillous deposition and morphological characteristics of maternal malpefusion (Hematoxylin-Eosin, Original Magnification 10×). (**B**) Detail of previous image shows a vessel with a thrombus in various stages of organization (Hematoxylin-Eosin, Original Magnification 20×).

An example of some of these placental changes is reported in Appendix A (Figure A1).

At immunohistochemistry, all sections of 29 placentas from SARS-CoV-2-positive mothers and of 28 placentas from control mothers were tested for anti-SARS-CoV-2 S1 spike protein immunostaining.

**Figure 1:**
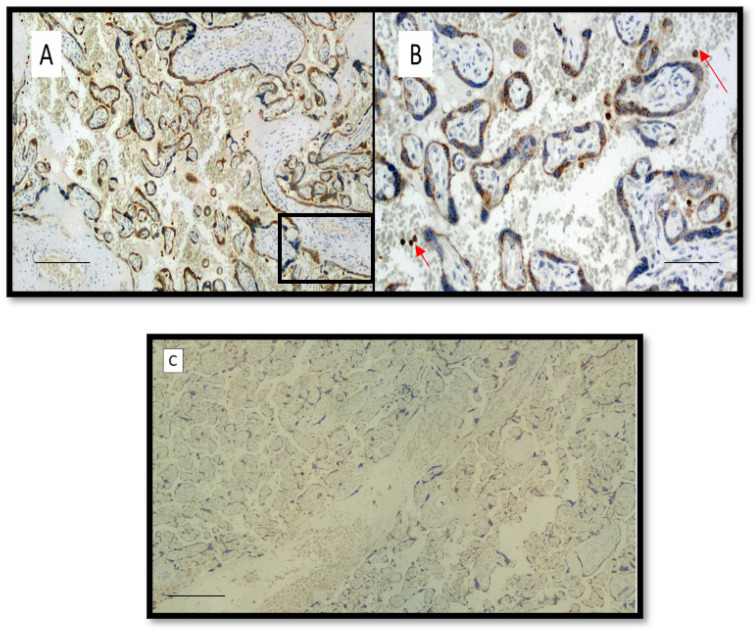
(**A**) Preparation for immunostaining with anti-SARS-CoV-2 S1 spike protein antibody in placentae from intensive care mothers (Immunohistochemistry, Original Magnification: 4×, scale bar: 700 µm). (**B**) Detail of box of previous picture of anti-SARS-CoV-2 S1 spike protein positivity (Immunohistochemistry, Original Magnification: 20×, scale bar: 350 µm) (red arrows indicate SARS-CoV-2-positive histiocytes). (**C**) Immunohistochemical negative control of a placenta from a negative SARS-CoV-2 mother (Immunohistochemistry, Original Magnification 4×, scale bar: 800 µm).

Figure 1 shows an example of preparation for immunostaining with anti-SARS-CoV-2 S1 spike protein antibody in placentae from intensive care mothers; note the intense positivity at the level, mainly, of the syncytiotrophoblast and of the intervillous histiocytes in the maternal space.

**Figure 2:**
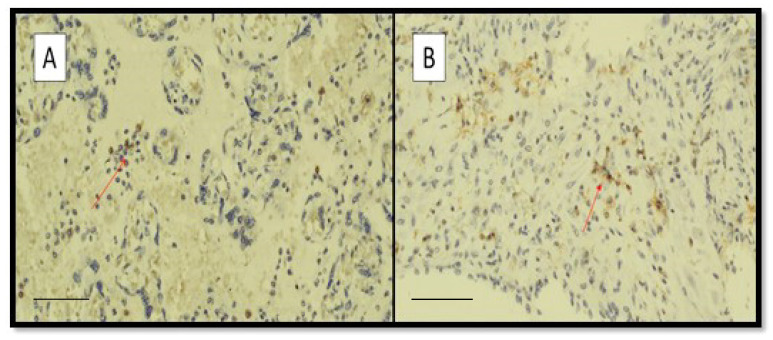
Examples of the presence of CD4 + T lymphocytes in intervillous space (**A**) and basal plate (**B**) from a mother belonging to CIC group. Note that, in our analysis, there was no statistically significant difference (*p* > 0.05) between the three groups. (Immunohistochemistry for CD4, Original Magnification 20×, red arrows indicate CD4 positive lymphocytes, scale bar: 350 µm).

Figure 2A,B shows examples of the presence of CD4 + T lymphocytes in intervillous space and basal plate from a mother belonging to CIC group.

**Figure 3:**
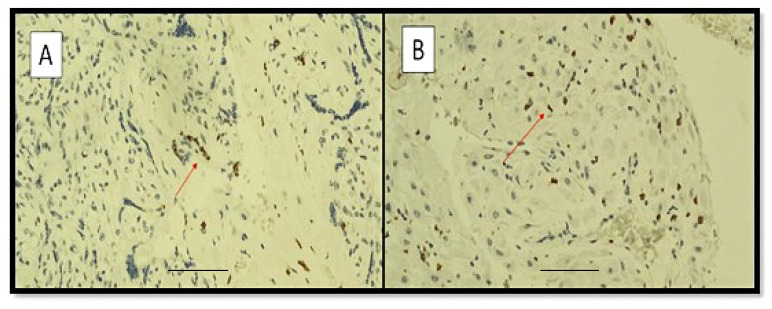
Examples of the presence of CD8 + T lymphocytes in intervillous space (**A**) and basal plate (**B**) from a mother belonging to CIC group. Note that, in our work, there was statistically significant difference only between CIC and control group but not between CIC and CNIC group. (Immunohistochemistry for CD8, Original Magnification 20×, red arrows indicate CD8 positive lymphocytes, scale bar: 350 µm).

Furthermore, Figure 3A,B shows examples of the presence of CD8 + T lymphocytes in intervillous space (A) and basal plate (B) from a mother belonging to CIC group.

**Figure 4:**
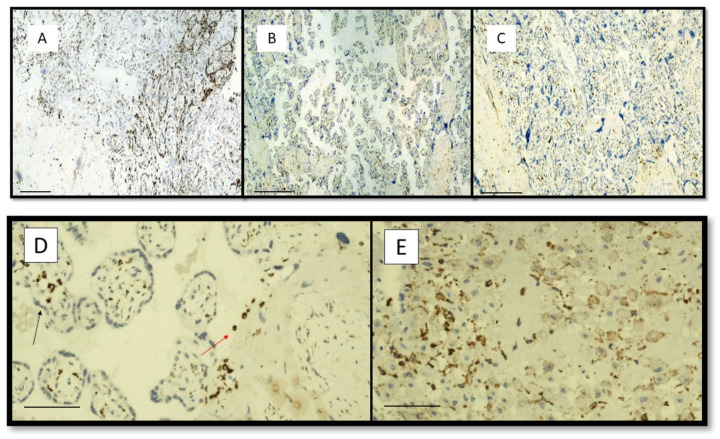
Examples of immunostaining with anti-CD68 antibody (PGM-1) in chorionic discs from CIC (**A**), CNIC (**B**) and control (**C**) groups. ((**A**), Original Magnification: 4×, scale bar: 700 µm. (**B**,**C**), Original Magnification: 10×, scale bar: 700 µm). (**D**) Photomicrograph showing the presence of CD68 positive histiocytes/macrophages in the intervillous space of a mother’s placenta belonging to the CIC group; note that the red arrow indicates the intervillous histocytes (those studied and analyzed in our work), while the black arrow indicates the Hofbauer cells (CD68 positive), which make up a population of cells residing within the chorionic villi. (**E**) Photomicrograph showing the presence of CD68 positive histiocytes/macrophages in the basal plate of a placenta from a mother belonging to the CIC group; note the extensive positivity (Immunohistochemistry for CD68, Original Magnification 40×). Scale bar: 500 µm.

Figure 4 shows examples of immunostaining with anti-CD68 antibody (PGM-1) in chorionic discs from CIC (A), CNIC (B) and control (C) groups; note the extensive positivity, more pronounced in the placentas from SARS-CoV-2-positive women of the CIC group with respect to the other groups. In Figure 4D, the presence of histiocyte–macrophage elements (CD68 positive) is appreciated both in the intervillous space and at the level of Hofbauer cells. Figure 4E shows the extensive and widespread presence of CD68 positive histiocytic elements at the level of the maternal decidua.

**Figure 5:**
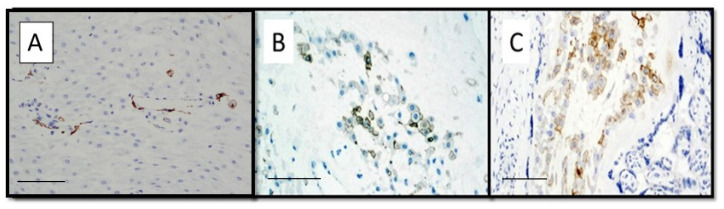
Examples of immunostaining with anti-ACE2 antibody in chorionic discs from the CIC (**A**), CNIC (**B**) and control (**C**) groups (Immunohistochemistry, Original Magnification: 20× (**A**–**C**). (scale bar: 400 µm).

Figure 5 shows examples of immunostaining with anti-ACE2 antibody in chorionic discs from the CIC (A), CNIC (B) and control (C) groups.

**Figure 6:**
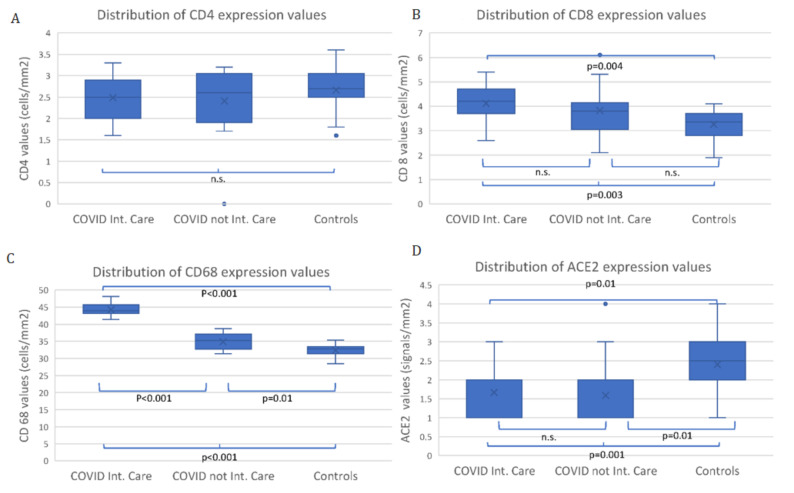
CD4 (**A**), CD8 (**B**), CD68 (**C**) and ACE2 (**D**) expression values in the COVID Int. Care (13 patients), COVID not Int. Care (16 patients) and Controls (28 patients).

CD4, CD8, CD68 and ACE2 expression values in the CIC, CNIC and control groups are represented in Figure 6A–D. No significant differences were observed among groups for CD4 (*p* > 0.05) (Figure 6A). A significant difference was observed in CD8 expression values between CIC and controls (*p* = 0.004) but not between CIC and CNIC (*p* > 0.05) (Figure 6B). CD68 expression values were significantly different among groups (*p* < 0.001), and the difference was significant in each pairwise comparison (Figure 6C). ACE2 expression values for CIC and CNIC were significantly lower vs. controls (*p* = 0.001) but not significantly different between the two SARS-CoV-2 groups (*p* > 0.05) (Figure 6D). In all three groups there was no immunolabeling at the level of extravillous trophoblasts (EVT) but only at the level of maternal decidua cells.

## 4. Discussion

Since the early months of the pandemic, interest in SARS-CoV-2-positive pregnant mothers has attracted the attention of the scientific community. Although in the first months only case reports and small case series were available for histopathological and immunohistochemical analysis, over the ensuing months, efforts to clarify the relationship between pregnancy, SARS-CoV-2 and possible neonatal outcomes multiplied considerably [14,15,16]. From the various studies presented in the literature, it would seem that the neonatal transmission rate of SARS-CoV-2 ranges between 0.5% and about 3%, and it is now quite clear that positivity for SARS-CoV-2 is distinct from placental infection, which is again different from vertical transmission [17,18]. No case of maternal–fetal transmission of SARS-CoV-2 was found in our study.

Various authors, including our group [7,8,14,15,16,17,18,19], have reported different histopathological patterns affecting the placental parenchyma in SARS-CoV-2-positive women. It seems that alterations, such as maternal thrombosis, deciduitis, increased intervillous fibrin, and, in rare cases, fetal thrombosis, are more commonly found in this patient population. Furthermore, there have already been previous attempts to correlate the severity of the maternal infection with potential placental histopathological changes; in fact, the concept of “viremia” was applied to try to correlate the amount of virus present in the circulation with the possible placental and vertical transmission of the infection [20]. From the studies examined, it seems that a greater viremia, and therefore symptoms, translates into a greater risk of vertical transmission and adverse outcomes [21,22]. In this work, carried out with the aim of identifying any differences in the histopathological patterns of placental damage among women in intensive care (with severe symptoms) and women not admitted to intensive care (asymptomatic or with mild symptoms) and the control group of healthy mothers from the historical archive, we found a statistically significant increased tendency of vascular maternal malperfusion in the CIC group compared to the CNIC and control groups, with a significant RR for CIC vs. CNIC. This finding was corroborated by a similar trend with regard to the deposition of perivillous fibrin, as if greater histological alterations were found as the viral load and viremia increased, also represented by an increased quantity of fibrin. Furthermore, the higher percentage of decidual arteriopathy and a higher frequency of thrombus formation in fetal blood vessels in the CIC group could be explained by the concept of “uncontrolled activation” of the coagulation cascade by SARS-CoV-2 (so-called storm cytokine) [23]. From the data in our possession and available in the literature, it would seem that a pathophysiological process occurs at the placental level, similar to what is described in the lung; Iba et al. have described in detail the events of endotheliopathy and coagulopathy that occur at the level of the beds’ vascular of arteries, veins and capillaries, leading to delineation of the so-called CAC, or both coagulopathies, from COVID-19 [24,25,26]. In their paper, Flores-Pliego et al. try to correlate the expression of some molecules (evaluated with immunofluorescence) to the degree of severity of the COVID-19 disease. The authors declare that the expression of the von Willebrand factor increases in the decidua endothelium and in the chorionic villi of the placenta derived from women with COVID-19, being higher in severe cases; Claudin-5 and VE-cadherin expression decreased in the decidua and chorionic villi of the placenta of women with severe COVID-19 but not in those with mild disease. Therefore, these data suggest that the placentas of women with COVID-19 have a permeable and thrombosed endothelial condition, sensitive to the severity of the disease [25].

Concerning the study of the immunological substrate in placentas, we found that CD4 + T cell density was entirely comparable in the three study groups, while there was a statistically significant difference in terms of CD8 + T lymphocytes in the CIC group compared to the control group (at the level of maternal decidua and the intervillous space). This finding could be explained by the possibility that placentas of COVID-positive mothers with severe symptoms are subject to a greater tendency of chronic villitis, as recently reported by Bertero L. et al. [27]. This picture could be integrated with a peripheral CD4 and CD8 immune response deficiency in patients with mild COVID-19 symptoms compared to patients with moderate/severe symptoms, as reported very recently by Bukowska-Ośko et al. [28].

Regarding the CD68 marker (PG-M1), the infiltration of intervillous macrophages (CD68+) in the placentas of mothers in the CIC group was higher compared to the CNIC and control groups. As also demonstrated by us in the previous work [7], it would seem that the sensitization of the histiocytes to the viral insult would increase the recall of the same in the intervillous space (it must be remembered that the Hofbauer cells are positive for CD68 but should not be counted in the analysis), thus supporting more the electron microscopy data that demonstrate the presence of virions inside the histiocytes parasitized by SARS-CoV-2. These data are in agreement with various studies in the literature [29,30,31,32,33,34], which reported a greater increase in macrophages, interpreted as an attempt to block and limit the spread of the virus or as a “side effect” of an inflammatory response on a dysregulated immune system basis.

Finally, regarding the immunohistochemical expression of ACE-2, an obligatory premise must be made. Bloise et al. [35] pointed out that ACE-2 and TMPRSS2 are expressed differently depending on the stage of pregnancy. In particular, they demonstrated that as the pregnancy progresses, a marked reduction in these two proteins occurs both at the level of the syncytiotrophoblast and at the smooth muscle, vascular endothelium and maternal decidua levels. These data could explain why, in our study, the immuno-expression was very low in general, and only a mild positivity was retained at the level of the cells constituting the maternal decidua. On the other hand, a recent paper by Bardon-Faure et al. seems to contradict this hypothesis, as these authors have described both a continuous positivity of ACE2 during pregnancy and a positive immunostaining of the immunohistochemistry in the placentas of SARS-CoV-2-positive mothers [36].

Another interpretation could be provided by the paper by Verma S. et al. [37]; in their series of cases, they describe a very reduced ACE2 immune expression in the placentas of SARS-CoV-2-positive mothers, thus suggesting the possibility of a true downregulation of the receptor in the course of viral infection. Our results seem to be in agreement with this last interpretation. However, it should be emphasized that these data need to be confirmed by protein quantification methods, such as the Western blot, as well as by immunohistochemistry, as performed in our study.

Finally, it seems quite certain that the low incidence rate of maternal–fetal transmission is due to a “barrier” effect, so that the placenta acts as the ultimate bulwark against SARS-CoV-2 [38,39,40]. Similarly, it does not seem that ACE-2, at least in the third trimester of pregnancy, is able to act as a “gateway” to virions but rather some other receptor, which has yet to be well characterized. From this point of view, one possibility could be transmembrane cellular protease serine 2 (TMPRSS2), which, being more strongly expressed than ACE-2, could act as a “gateway” to SARS-CoV-2 endocytosis [39,41].

Regarding preterm births in the three groups, there is a significant prevalence of early birth in the SARS-CoV-2-positive groups with respect to control group. Our data further confirm different previous works that agree in counting preterm birth among the adverse effects of SARS-CoV-2 infection [40,42,43]. Furthermore, Blitz et al., in a recent paper [44], clearly demonstrate that women with severe SARS-CoV-2 symptoms are at greater risk of premature birth of the newborn than cases of asymptomatic infections.

### Limitations

The present work was focused on studying the distribution of immune cells on formaldehyde-fixed and paraffin-embedded (FFPE) samples, a process that may have created a “wash-out” of cells at the intervillous level. In addition, the study evaluated a limited number of placentas, and the control group was randomly selected from the historical archive.

Furthermore, it was not possible to obtain and correlate the histological and immunophenotypic data with the ultrasound data, nor to study the profile of maternal leukocytes. It should also be clarified that it is possible that intensive care per se could have repercussions on the placentas.

## 5. Conclusions

There is still much to study and discover regarding the etiopathogenesis mechanisms of SARS-CoV-2; there is even more to understand about immunopathology and the relationship between placental parenchyma, virus and host response. Pregnant intensive care patients show severe alterations of the placenta, which might negatively affect its ability to support the fetus. Therefore, placental function needs to be closely monitored in the affected mothers. Whether or not placental malfunction, as indicated by IHC, might lead to preterm birth, as indicated by our findings, remains to be prospectively examined in a larger cohort.

## Figures and Tables

**Table 1:** Maternal and pregnancy features.

Maternal and Pregnancy Features	COVID Intensive Care (13 Mothers)	COVID non Intensive Care (16 Mothers)	Control Group (28 Mothers)	*p* Values
Gestational age (week), means ± sd	35.2 ± 4.4	36.9 ± 1.8	38 ± 2	0.020
Preterm, *n* (%)	7 (53.8)	9 (56.2)	4 (14.3)	0.005
Primiparous, *n* (%)	6 (50.0) •	6 (40.0) •	17 (60.7)	0.420
Cesarean Section, *n* (%)	9 (81.8) ••	10 (71.4) • §	26 (92.8)	0.180

• presence of 1 missing data, •• presence of 2 missing data, § presence of 1 IVG.

**Table 2:** Placental findings in COVID intensive care, COVID not intensive care and control groups.

Placental Finding	COVID Intensive Care (13 Placentae)	COVID non Intensive Care (16 Placentae)	Control Group (28 Placentae)	FDR Corrected *p* Values	OR (95%CI)
Weight (grams), means ± sd	445.8 ± 104.9	547.2 ± 100	533 ± 188	0.02	-
Maternal malperfusion, *n* (%)	13 (100.0)	11 (68.7)	11 (39.3)	0.001	1.5 (1.1–2.2) §
Decidual arteriopathy, *n* (%)	12 (92.3)	7 (43.7)	1 (3.6)	<0.001	15.4 (1.6–148.8)
Fetal malperfusion, *n* (%)	6 (46.2)	6 (37.5)	0	0.002	1.4 (0.3–6.3)
Decidual inflammation, *n* (%)	11 (84.6)	10 (62.5)	0	<0.001	3.3 (0.5–20.3)
Perivillous fibrin deposition, *n* (%)	12 (100.0) •	11 (68.7)	3 (11)	<0.001	1.4 (1–2) §
Terminal villous hyperplasia *n* (%)	8 (61.5)	3 (18.8)	5 (17.9)	0.01	6.9 (1.3–37.2)
Villous hypervascularization, *n* (%)	4 (30.8)	2 (12.5)	12 (42.9)	0.11	3.1 (0.5–20.6)
Thrombi in fetal vessels, *n* (%)	12 (92.3)	5 (31.3)	0	<0.001	26.4 (2.6–262)
Syncytial nodes, *n* (%)	2 (15.4)	5 (31.3)	14 (50.0)	0.10	0.4 (0.06–2.5)
Chorioamnionitis, *n* (%)	1 (7.7)	4 (25.0)	0	0.02	0.25 (0.02–2.6)
Perivillary histiocytosis, *n* (%)	11 (84.6)	9 (56.3)	5 (17.9)	<0.001	4.3 (0.7–25)

O.R. or RR(§) were calculated to evaluate the risk of specific placental findings in CIC vs. CNIC’; FDR correction refers to comparisons of proportions and RR results. • presence of 1 missing data.

## Data Availability

Not applicable.
